# Comparison of the ability of different quantitative indices in ^123^I-FP-CIT single-photon emission computed tomography to differentiate dopaminergic neurodegenerative disease

**DOI:** 10.1007/s11604-024-01648-7

**Published:** 2024-09-05

**Authors:** Tomohiro Sato, Setsu Sawai, Naokazu Shimada

**Affiliations:** 1 Department of Radiology, University of Tokyo Hospital, 7-3-1 Hongo, Bunkyo-ku, Tokyo, 113-8655 Japan; 2 Department of Radiology, Chiba Aoba Municipal Hospital, 1273-2 Aoba-cho, Chuo-ku, Chiba City, Chiba 260-0852 Japan; 3 Department of Neurology, Chiba Aoba Municipal Hospital, 1273-2 Aoba-cho, Chuo-ku, Chiba City, Chiba 260-0852 Japan

**Keywords:** ^123^I-FP-CIT, DVR, SBR, SUV, Parkinson’s disease

## Abstract

**Purpose:**

By imaging dopamine transporter (DAT) uptake in the striatum, ^123^I-FP-CIT SPECT can differentiate dopaminergic neurodegenerative disease (dNDD) and non-dNDD, which differ in pathophysiology and clinical management. Our aim was to compare and validate the diagnostic abilities of various ^123^I-FP-CIT SPECT quantitative indices for dNDD.

**Materials and methods:**

Distribution volume ratio (DVR) and binding ratio (BR), measures of DAT uptake capacity, were measured by analyzing clinical ^123^I-FP-CIT SPECT images of 29 patients with dNDD, including dementia with Lewy bodies and Parkinson’s disease, and 18 patients with non-dNDD, using Montreal Neurological Institute space-based anatomical standardization and an atlas template, which utilizes statistical parametric mapping. Additionally, we computed the specific binding ratio (SBR) based on Bolt’s method and the maximum and mean standardized uptake values (SUVmax and SUVmean, respectively).

**Results:**

The caudate-to-occipital lobe, putamen-to-occipital lobe, and striatum-to-occipital lobe ratios (COR, POR, and SOR, respectively) on DVR and POR and SOR on BR were significantly lower in dNDD than in non-dNDD, with areas under the ROC curve (AUCs) of 0.941–0.960, showing high diagnostic accuracy for dNDD. However, the AUC of COR on BR was 0.839, indicating lower diagnostic performance. SBR had an AUC of 0.921, while SUVmax and SUVmean had AUCs of 0.906 and 0.900, respectively. Although striatal asymmetry on both DVR and BR exhibited AUCs of 0.728 and 0.734 and asymmetry on SBR showed an AUC of 0.757, the ratio-based DAT quantitative indices were superior. There were strong positive correlations of DVR with BR, DVR with SBR or SUVmax, BR with SBR or SUVmax, and SBR with SUVmax.

**Conclusion:**

COR, POR, and SOR on DVR and POR and SOR on BR were the most useful DAT quantitative indices. These indices can be compared with SBR and SUV, suggesting that comprehensive evaluation improves the diagnostic accuracy of dNDD.

## Introduction

Dementia with Lewy bodies (DLB) and Parkinsonʼs disease (PD) are nigrostriatal dopaminergic neurodegenerative diseases (dNDDs) characterized by reduced expression of dopamine transporter (DAT) in striatal dopamine nerve terminals [1–3]. The diagnostic sensitivity of DLB is low, and it is often confused with Alzheimer’s disease (AD) in the absence of Parkinsonism [2, 3]. Indeed, many patients with DLB also have pathological findings of AD [4]. Although antipsychotic treatment is effective for the psychiatric symptoms of AD, PD and DLB are at risk of worsening extrapyramidal symptoms and impaired activities of daily living due to hypersensitivity reactions [3, 5], which are also associated with increased mortality [2]. Accurate differentiation of PD and DLB from AD is important for effective drug therapy and avoiding neuroleptic hypersensitivity [6]. PD is assessed by evaluating motor and non-motor symptoms as well as their severity according to Movement Disorder Society Unified Parkinson’s Disease Rating Scale (MDS-UPDRS) and Hoehn and Yahr stage (HY stage) [1, 7], which are used to review treatment and nursing care. In PD, levodopa treatment slows the progression of motor symptoms and improves prognosis [8, 9], and PD must be distinguished from essential tremor (ET) and drug-induced Parkinsonism (DIP), which present with similar clinical symptoms, because their clinical management is different [10, 11].

^123^I-2β-carbomethoxy-3β-(4-iodophenyl)-N-(3-fluoropropyl) nortropane (^123^I-FP-CIT) single-photon emission computed tomography (SPECT) can image DAT to differentiate dNDD and non-dNDDs, such as AD, ET, and DIP, which differ in pathophysiology and clinical management [10–13]. The ^123^I-FP-CIT SPECT diagnostic method includes visual determination of striatal DAT uptake and objective evaluation using quantitative indices and, when combined, improves the diagnostic accuracy of dNDD [14]. One quantitative technique involves a manual depiction of regions of interest (ROIs) in the striatum and background and measurement of the γ-ray count ratio [12], but the quantitative value of the striatum is underestimated due to the partial volume effect caused by the low resolution of SPECT images [15]. Therefore, researchers have proposed a specific binding ratio (SBR) based on Bolt’s method, which reduces the partial volume effect by setting a larger ROI than the target striatum and calculates the ratio of the specific binding concentration in the striatum to the brain parenchymal background [14, 16].

Other quantitative methods include DAT uptake and ratios in the striatum divided into the caudate and putamen, uptake ratios in the left/right cerebral hemispheres [13, 17, 18], automated measurements using Montreal Neurological Institute (MNI) spatial-based anatomical standardization and the atlas template ROI, which utilizes statistical parametric mapping (SPM) [19–21], and the application of standardized uptake value (SUV), which is used in positron emission tomography (PET) to determine cell metabolism [22–24].

Few reports have compared all of the available striatal DAT quantification methods. Furthermore, new software to calculate DAT uptake in the caudate and putamen recently became available in Japan, but its clinical utility requires verification. Here, we compared and evaluated the abilities of different ^123^I-FP-CIT SPECT quantitative indices to differentiate dNDD from non-dNDD.

## Materials and methods

### Patients

^123^I-FP-CIT SPECT was performed in 47 patients with suspected dNDD from June 2020 to August 2023. A comprehensive diagnosis was made by a neurologist based on patients’ cognitive impairment, psychiatric symptoms, motor dysfunction, HY stage, and imaging tests, such as computed tomography (CT), magnetic resonance imaging, and nuclear medicine except ^123^I-FP-CIT SPECT. Inclusion criteria were clinically established PD or clinically probable PD according to MDS-UPDRS [1] and probable DLB according to the Neurology criteria [2]. All PD patients showed good levodopa responsiveness.

The clinical diagnoses were 29 patients with dNDD, including 25 patients with PD and 4 patients with DLB, and 18 patients with non-dNDD, including 5 patients with ET, 5 patients with DIP, 3 patients with AD, and 5 patients with other diseases, such as depression and cervical spondylosis. Of the 21 PD and 2 DLB cases with a severity diagnosis, 5 were HY stage 1, 10 were HY stage 2, and 8 were HY stage 3. There were no cases of reduced DAT uptake in the striatum or parenchyma due to organic disease.

This retrospective observational study was conducted with anonymized personal information and was approved by the ethics committee of Chiba Aoba Municipal Hospital (approval number 0503 [2023]). Moreover, patients were given the opportunity to opt out via a notice posted in the institution.

### Imaging protocol for 123I-FP-CIT SPECT/CT

SPECT/CT scans were performed using a dual-head SPECT/CT system with low-energy high-resolution collimators and 6-row detector CT (Symbia Intevo6; Siemens Healthineers, Erlangen, Germany) 4 h after intravenous injection of 167 MBq of ^123^I-FP-CIT (Nihon Medi-Physics Co., Ltd., Tokyo, Japan).

SPECT scans were acquired with 2.4-mm pixels in a 256 × 256 matrix and 90 projections over 360° in a circular orbit of radius 14 cm with a dwell time of 40 s/frame (i.e., total acquisition time of 30 min). The energy windows were set at 159.0 keV ± 7.5% for the photopeak window and at 176.5 keV ± 3.5% and 141.5 keV ± 3.5% for the sub-window. After the SPECT acquisition, CT scans were acquired using a tube voltage of 130 kVp, detector collimation of 6 × 2.0 mm, an automatic exposure control algorithm with 100 quality reference mAs (CARE Dose 4D; Siemens Healthineers), a gantry rotation time of 1.5 s, and a pitch factor of 1.0. CT data were reconstructed with a 3.0 mm slice thickness and 300 mm field of view using a filter kernel (H31s medium; Siemens Healthineers).

SPECT data were reconstructed using a workstation (Syngo MI Applications VB20 software; Siemens Healthineers) with ordered-subset conjugate gradient minimization (OSCGM) algorithms with scatter correction using a dual-energy window approach, attenuation correction based on a CT-derived attenuation map, and depth-dependent spatial resolution correction. The reconstruction parameters were set to 1 subset and 30 iterations and a Gaussian filter with a full-width at half-maximum (FWHM) of 7.2 mm with examination type “Brain”. SPECT images were adjusted for the cerebral axis by referring to the CT images so that the transverse plane was parallel to the anterior commissure-posterior commissure line.

Striatal DAT quantitative values depend on the reconstruction parameters, and correction for scattering, attenuation, and spatial resolution reduces errors from the true value and improves accuracy [25, 26]. Therefore, we validated the optimal iterations and FWHM of the Gaussian filter in ^123^I-FP-CIT SPECT with application of all compensations, through preliminary physical evaluation of striatal contrast, background noise, and recovery coefficient using a striatal phantom.

### Sensitivity calibration procedure for SPECT quantification

The SPECT/CT system was calibrated by planar and volume sensitivity resulting from periodic planar scanning of a syringe containing 167 MBq ^123^I and SPECT scanning of a cylinder phantom with a 6380 mL volume containing 40 MBq ^123^I, using a quantification application (Broad Quantification; Siemens Healthineers) and workstation. The actual radioactivity was measured using a dose calibrator (CRC-55t; Capintec Inc., Florham Park, NJ) and the mean count in the voxel of interest (VOI) drawn on the SPECT image was measured. The Becquerel calibration factor (BCF) is calculated using Eq. 1.

1 $$\text{BCF }=\frac{\text{actual radioactivity}/\text{phantom volume}}{\text{mean count in the VOI}}$$

The radiation decay was compensated by the recorded times of the radioactivity measurement and the scans. Consequently, voxel values displayed on the workstation were converted from the γ-ray count to radioactivity concentration (Bq/mL) by the sensitivity calibration, allowing the below-mentioned SUV measurements.

### Imaging analysis for DAT quantitative indices

**Fig. 1:**
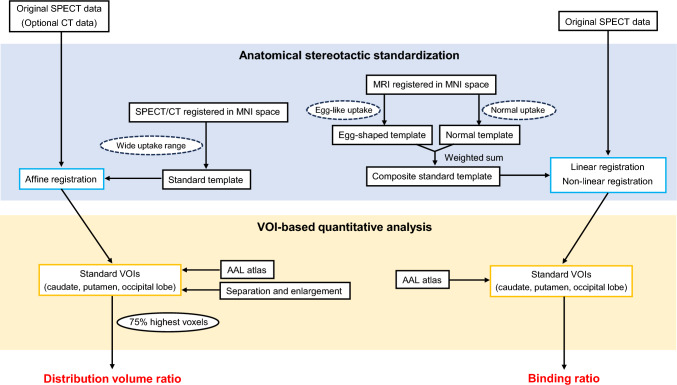
Overview of processing techniques for DVR and BR

The following striatal DAT quantitative indices were calculated by analyzing clinical SPECT/CT images. Distribution volume ratio (DVR) of DAT uptake in the caudate and putamen was measured using Scenium software (Siemens Healthineers). Definition of the DVR here is provided in Appendix, Table 1. The analytical procedure for DVR is shown in Fig. 1. First, an original ^123^I-FP-CIT SPECT image or optional CT image was input into the software and then affine registered to the standard template. The standard template was created as follows: (1) affine registration of an individual ^123^I-FP-CIT SPECT/CT image to MNI space using SPM5, deformation of the SPECT image with the resulting parameters, and normalization of the voxels by the mean uptake value in the occipital lobe; and (2) affine registration of SPECT images in diverse ^123^I-FP-CIT uptake groups to the normalized image and additive averaging.

**Fig. 2:**
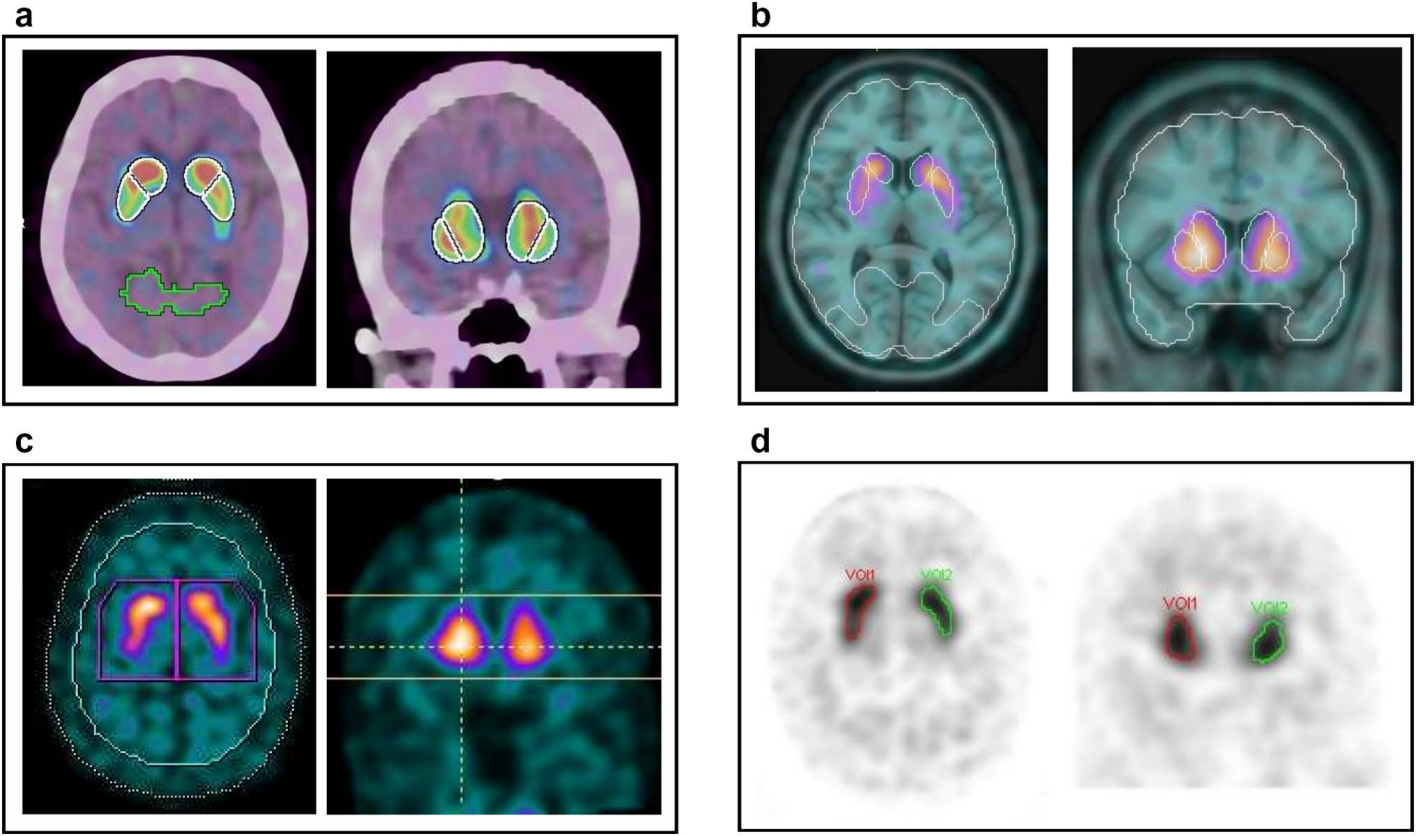
Outline of VOIs on striatal SPECT images using different methods for quantitative analysis. **a** DVR, **b** BR, **c** SBR, and **d** SUV

Then, standard VOIs were depicted on the caudate, putamen, and occipital lobes in the original SPECT image registered to the standard template. The outline of the VOI in the DVR analysis is shown in Fig. 2a. A standard VOI was created by (1) segmenting the striatum from the standard template based on the automated anatomical labeling (AAL) atlas [19, 26]; and (2) separating the striatum into the caudate and putamen with slight enlargement, giving caudate and putamen volumes of 11 and 9 mL, respectively. The caudate-to-occipital lobe, putamen-to-occipital lobe, and striatum-to-occipital lobe ratios (COR, POR, and SOR, respectively) and the putamen-to-caudate ratio (PCR) were calculated using Eqs. 2–5.

2 $$\text{COR on DVR}=\frac{\text{mean count in the }75\text{ percent highest voxels in the caudate VOI}}{\text{mean count in the occipital lobe VOI}}$$

3 $$\text{POR on DVR}=\frac{\text{mean count in the }75\text{ percent highest voxels in the putamen VOI}}{\text{mean count in the occipital lobe VOI}}$$

4 $$\text{SOR on DVR}=\frac{\text{mean count in the }75\text{ percent highest voxels in the striatum VOI }}{\text{mean count in the occipital lobe VOI}}$$

5 $$\text{PCR on DVR}=\frac{\text{mean count in the }75\text{ percent highest voxels in the putamen VOI}}{\text{mean count in the }75\text{ percent highest voxels in the caudate VOI}}$$

Additionally, index asymmetries on DVR, which represent left/right differences in DAT uptake, were calculated using Eq. 6.

6 $$\text{Asymmetry on DVR }\left(\text{\%}\right)=\frac{ \left|{\text{DVR}}_{\text{right}} - {\text{DVR}}_{\text{left}}\right|}{{(\text{DVR}}_{\text{right}} + {\text{DVR}}_{\text{left}})/2 }\times 100 (\%)$$

Binding ratio (BR) was measured using DaTView software (Nihon Medi-Physics Co., Ltd.), a recently updated software package. Definition of the BR here is provided in Appendix, Table 1. The analytical procedure for BR is also shown in Fig. 1. First, the original ^123^I-FP-CIT SPECT image was input into the software, and then linear and non-linear registration were performed to the standard template formed by weighted averaging of the normal and egg-shaped templates by the adaptive atlas for adaptation to the target [27]. A standard template was created by (1) registering ^123^I-FP-CIT SPECT and brain MRI images of the same individual, using diffeomorphic anatomical registration through exponentiated lie algebra (DARTEL) implemented in SPM8; (2) registering the brain MRI image to MNI space, deforming the SPECT image with the resulting parameters, and normalizing the voxels by the mean value of the uptake in the occipital lobe; and (3) registering SPECT images in the normal and egg-like ^123^I-FP-CIT uptake groups to the normalized image and additive averaging.

Then, standard VOIs based on the AAL atlas [19, 26] were depicted on the caudate, putamen, and occipital lobes in the original SPECT image registered to the standard template. The outline of the VOI in the BR analysis is shown in Fig. 2b. COR, POR, SOR, and PCR were calculated using Eqs. 7–10.

7 $$\text{COR on BR}=\frac{\text{mean count in the caudate VOI}}{\text{mean count in the occipital lobe VOI}}-1$$

8 $$\text{POR on BR}=\frac{\text{mean count in the putamen VOI}}{\text{mean count in the occipital lobe VOI}}-1$$

9 $$\text{SOR on BR}=\frac{\text{mean count in the striatum VOI}}{\text{mean count in the occipital lobe VOI}}-1$$

10 $$\text{PCR on BR}=\frac{\text{mean count in the putamen VOI}}{\text{mean count in the caudate VOI}}$$

Additionally, index asymmetries on BR were calculated using Eq. 11.

11 $$\text{Asymmetry on BR }\left(\text{\%}\right)=\frac{ \left|{\text{BR}}_{\text{right}} - {\text{BR}}_{\text{left}}\right|}{{(\text{BR}}_{\text{right}} + {\text{BR}}_{\text{left}})/2 }\times 100 (\%)$$

The abovementioned SBR based on Bolt’s method was measured using DaTView software [16]. Definition of the SBR is provided in Appendix, Table 1. Large VOIs with a slab thickness of 44.5 mm were automatically depicted at the center of the striatum and in the background. The outline of the VOI in the SBR analysis is shown in Fig. 2c. The background contour was extracted using the distance-weighted histogram method set to a FWHM of 16 mm, and the assumed striatum volume was set to 11.2 mL. SBR and its asymmetry were calculated using Eqs. 12 and 13.

12 $$\text{SBR}=\frac{\frac{\text{total count in the striatum VOI }}{\text{count per unit volume in the background VOI}} - \text{volume of striatum VOI}}{\text{striatum volume}}$$

13 $$\text{Asymmetry on SBR }\left(\text{\%}\right)=\frac{ \left|{\text{SBR}}_{\text{right}} - {\text{SBR}}_{\text{left}}\right|}{{(\text{SBR}}_{\text{right}} + {\text{SBR}}_{\text{left}})/2 }\times 100 (\%)$$

Striatal SUV was measured using a workstation. First, VOIs were drawn and modified manually around the visible bilateral striatum. Then, the VOIs were semi-automatically adjusted to fit the striatal uptake based on the threshold maximum voxel value, defined as 65%. The outline of the VOI in the SUV analysis is shown in Fig. 2d. SUVmax, SUVmean, and their asymmetries were calculated using Eqs. 14–17.

14 $$ SUV\max = \frac{\max\,\,voxel\,\,values\,\,in\,\,the\,\,striatum\,\,VOIs}{{administration\,\,dose/patient{\text{'}}s\,\,weight}} $$

15 $$ SUVmean = \frac{mean\,\,voxel\,\,values\,\,in\,\,the\,\,striatum\,\,VOIs}{{administration\,\,dose/patient{\text{'}}s\,\,weight}} $$

16 $$\text{Asymmetry on SUVmax }\left(\text{\%}\right)=\frac{ \left|{\text{SUVmax}}_{\text{right}} - {\text{SUVmax}}_{\text{left}}\right|}{{(\text{SUVmax}}_{\text{right}} + {\text{SUVmax}}_{\text{left}})/2 }\times 100 (\%)$$

17 $$\text{Asymmetry on SUVmean }\left(\text{\%}\right)=\frac{ \left|{\text{SUVmean}}_{\text{right}} - {\text{SUVmean}}_{\text{left}}\right|}{{(\text{SUVmean}}_{\text{right}} + {\text{SUVmean}}_{\text{left}})/2 }\times 100 (\%)$$

### Statistical analysis

Statistical analysis was performed using EZR (Saitama Medical Center, Jichi Medical University, Saitama, Japan), a graphical user interface for R (R Development Core Team, Vienna, Austria) [28]. Differences in patients’ characteristics and DAT quantitative values between dNDD and non-dNDD were assessed by Fisher’s exact test for nominal variables and the *t* test and Welch test for continuous variables. Correlations in quantitative indices were assessed by Pearson product-moment correlation coefficients. In addition, differences in disease duration and quantitative values between H–Y stages were assessed using the Steel–Dwass test. *P* < 0.01 was considered statistically significant. Normality and equal variances of the data regarding the choice of statistical method were ascertained by Kolmogorov–Smirnov and F tests. Diagnostic accuracy for dNDD was determined by receiver operating characteristic (ROC) analysis. Optimal cutoff, sensitivity, and specificity were defined by Youden’s index.

## Results

### Differences in DAT quantitative values between dNDD and non-dNDD

Sex, age, and DAT quantitative indices of dNDD and non-dNDD patients are shown in Table 2, with DAT quantitative values defined as the affected side with low uptake. All data except sex are expressed as means and standard deviations.

**Table 2:** Comparison of patient characteristics and DAT quantitative indices with different analysis methods between dNDD and non-dNDD. DAT quantitative values are defined as lower striatum uptake side

Variable	dNDD	non-dNDD	*P* value
Sex (F/M)	11/18	13/5	0.0355
Age (years)	76.1 ± 6.7	73.5 ± 6.8	0.204
COR on DVR	2.81 ± 0.65	4.37 ± 0.66	< 0.01
POR on DVR	2.18 ± 0.59	3.89 ± 0.72	< 0.01
SOR on DVR	2.57 ± 0.62	4.20 ± 0.67	< 0.01
PCR on DVR	0.744 ± 0.055	0.869 ± 0.073	< 0.01
COR on BR	0.782 ± 0.338	1.39 ± 0.48	< 0.01
POR on BR	1.33 ± 0.58	2.88 ± 0.60	< 0.01
SOR on BR	1.13 ± 0.48	2.32 ± 0.57	< 0.01
PCR on BR	1.18 ± 0.14	1.38 ± 0.19	< 0.01
SBR	2.89 ± 1.23	5.52 ± 1.41	< 0.01
SUVmax	6.70 ± 1.73	10.5 ± 2.8	< 0.01
SUVmean	5.32 ± 1.39	8.31 ± 2.18	< 0.01
Caudate asymmetry on DVR	7.41 ± 4.94	3.47 ± 2.18	< 0.01
Putamen asymmetry on DVR	10.6 ± 7.8	6.85 ± 6.53	0.0831
Striatum asymmetry on DVR	6.97 ± 4.97	3.43 ± 2.72	< 0.01
Caudate asymmetry on BR	27.2 ± 16.0	30.4 ± 12.1	0.435
Putamen asymmetry on BR	17.9 ± 11.2	11.1 ± 5.9	< 0.01
Striatum asymmetry on BR	11.8 ± 9.9	5.21 ± 5.48	< 0.01
Asymmetry on SBR	21.1 ± 17.6	8.45 ± 6.08	< 0.01
Asymmetry on SUVmax	9.32 ± 8.22	5.01 ± 4.53	0.0253
Asymmetry on SUVmean	8.89 ± 8.77	4.82 ± 4.16	0.0382

*DAT* dopamine transporter, *dNDD* dopaminergic neurodegenerative disease, *F* female, *M* male, *DVR* distribution volume ratio, *BR* binding ratio, *COR* caudate-to-occipital lobe ratio, *POR* putamen-to-occipital lobe ratio, *SOR* striatum-to-occipital lobe ratio, *PCR* putamen-to-caudate ratio, *SBR* specific binding ratio, *SUV* standardized uptake value, *SUVmax* maximum SUV, *SUVmean* mean SUV

Sex was not associated with type of disease group, and age was not significantly different between disease groups. Meanwhile, COR, POR, SOR, and PCR on DVR were significantly lower in dNDD than in non-dNDD (2.81, 2.18, 2.57, and 0.744 vs. 4.37, 3.89, 4.20, and 0.869, respectively; *P* < 0.01). The quantitative indices on BR were also significantly lower in dNDD than in non-dNDD (COR, POR, SOR, and PCR: 0.782, 1.33, 1.13, and 1.18 vs. 1.39, 2.88, 2.32, and 1.38, respectively; *P* < 0.01). Moreover, COR, POR, and SOR were lower on BR than on DVR, while COR was higher than POR on DVR but lower than POR on BR. SBR, SUVmax, and SUVmean were also significantly lower in dNDD than in non-dNDD (2.89, 6.70, and 5.32 vs. 5.52, 10.5, and 8.31, respectively; *P* < 0.01). The disease duration and DAT quantitative indices for each HY stage are shown in Table 3. The disease duration was longer for HY stage 2 than for HY stage 1, but shorter for HY stage 3 than for HY stage 2; none of the differences was significant. COR, POR, SOR, and PCR on DVR were slightly lower for HY stage 2 than for HY stage 1, but were comparable for HY stage 2 and HY stage 3. Although COR, POR, and SOR on BR were lower for HY stage 2 than for HY stage 1, they were higher for HY stage 3 than for HY stage 2. PCR on BR did not change regardless of HY stage. SBR, SUVmax and SUVmean were lower for HY stage 2 than for HY stage 1, and even lower for HY stage 3 than for HY stage 2. However, there were no significant differences in any of the quantitative indices between HY stages.

**Table 3:** Comparison of disease duration and DAT quantitative indices with different analysis methods between HY stages. DAT quantitative values are defined as lower striatum uptake side

Variable	HY I	HY II	HY III	*P* value		
				I vs. II	I vs. III	II vs. III
Number of dNDD	5	10	8		NR	
Disease duration (months)	6.40 ± 2.97	26.3 ± 22.9	14.3 ± 19.1	0.052	0.741	0.190
COR on DVR	2.93 ± 0.42	2.73 ± 0.41	2.72 ± 0.59	0.779	0.744	0.996
POR on DVR	2.22 ± 0.42	2.07 ± 0.34	2.08 ± 0.50	0.967	0.827	0.962
SOR on DVR	2.63 ± 0.42	2.49 ± 0.37	2.49 ± 0.55	0.846	0.828	0.983
PCR on DVR	0.754 ± 0.037	0.730 ± 0.039	0.731 ± 0.076	0.434	0.953	0.948
COR on BR	0.992 ± 0.243	0.648 ± 0.210	0.826 ± 0.295	0.0704	0.654	0.246
POR on BR	1.54 ± 0.42	1.14 ± 0.26	1.23 ± 0.51	0.200	0.385	1.00
SOR on BR	1.34 ± 0.34	0.972 ± 0.232	1.08 ± 0.41	0.0935	0.471	0.897
PCR on BR	1.12 ± 0.14	1.16 ± 0.14	1.15 ± 0.13	0.813	0.899	0.962
SBR	3.21 ± 1.06	2.74 ± 0.99	2.47 ± 1.18	0.876	0.561	0.808
SUVmax	7.20 ± 1.30	6.87 ± 2.01	6.22 ± 1.72	0.967	0.561	0.897
SUVmean	5.73 ± 0.92	5.50 ± 1.68	4.88 ± 1.34	0.813	0.385	0.757
Caudate asymmetry on DVR	8.47 ± 5.22	5.13 ± 4.06	10.7 ± 5.9	0.438	0.988	0.0834
Putamen asymmetry on DVR	14.6 ± 3.5	9.80 ± 8.43	14.5 ± 8.2	0.306	0.988	0.377
Striatum asymmetry on DVR	10.8 ± 4.5	4.79 ± 3.47	10.6 ± 4.7	0.0935	0.988	0.0210
Caudate asymmetry on BR	26.7 ± 20.9	30.3 ± 17.8	20.7 ± 14.3	0.928	0.954	0.377
Putamen asymmetry on BR	17.9 ± 11.7	16.4 ± 10.0	23.0 ± 13.8	1.000	0.899	0.480
Striatum asymmetry on BR	13.6 ± 8.8	8.74 ± 4.82	15.5 ± 13.4	0.590	1.000	0.591
Asymmetry on SBR	18.9 ± 11.3	22.9 ± 19.7	27.5 ± 20.3	0.992	0.954	0.897
Asymmetry on SUVmax	11.4 ± 8.6	6.42 ± 7.88	8.29 ± 4.38	0.249	0.899	0.246
Asymmetry on SUVmean	10.4 ± 8.6	6.32 ± 8.40	7.99 ± 4.49	0.369	0.988	0.286

*DAT* dopamine transporter, *dNDD* dopaminergic neurodegenerative disease, *HY* Hoehn and Yahr stage, *DVR* distribution volume ratio, *BR* binding ratio, *COR* caudate-to-occipital lobe ratio, *POR* putamen-to-occipital lobe ratio, *SOR* striatum-to-occipital lobe ratio, *PCR* putamen-to-caudate ratio, *SBR* specific binding ratio, *SUV* standardized uptake value, *SUVmax* maximum SUV, *SUVmean* mean SUV, *NR* not reported

The asymmetries of all DAT quantification values, except for caudate asymmetry on BR, were higher in dNDD than in non-dNDD. In contrast, caudate asymmetry on BR was maximal and higher in non-dNDD than in dNDD (30.4 vs. 27.2). There were significant differences between dNDD and non-dNDD in caudate and striatal asymmetry on DVR, putamen and striatal asymmetry on BR, and asymmetry on SBR (*P* < 0.01). However, there were no significant differences in putamen asymmetry on DVR, caudate asymmetry on BR, and asymmetry on both SUVmax and SUVmean. The asymmetries of all DAT quantification values, except for caudate asymmetry on BR and asymmetry on SBR, were higher for HY stage 3 than for HY stage 2, but were lower for HY stage 2 than for HY stage 1. In contrast, caudate asymmetry on BR was higher for HY stage 2 than for HY stage 1, but was lower for HY stage 3 than for HY stage 2. Asymmetry on SBR became higher as the HY stage progressed. However, there were no significant differences in any of the quantitative asymmetries between the HY stages.

### Ability of DAT quantitative indices to differentiate dNDD

The diagnostic accuracy of the DAT quantitative indices and their asymmetries in dNDD are shown in Tables 4, 5. dNDD was identified when the DAT quantitative index was below the cutoff and the asymmetry was above the cutoff. COR, POR, and SOR on DVR showed very high diagnostic accuracy, while PCR was slightly inferior (area under the ROC curve [AUC] of 0.946 for COR and 0.960 for both POR and SOR vs. 0.908 for PCR). POR and SOR on BR also showed very high diagnostic accuracy, while COR and PCR were somewhat inferior (AUCs of 0.954 for POR and 0.941 for SOR vs. 0.839 for COR and 0.807 for PCR).

**Table 4:** Diagnostic accuracy of DAT quantitative indices for dNDD according to ROC analysis

	DVR				BR				SBR	SUVmax	SUVmean
	COR	POR	SOR	PCR	COR	POR	SOR	PCR			
Cutoff	3.70	2.62	2.88	0.790	1.34	2.05	1.42	1.25	3.57	9.09	6.88
Sensitivity (%)	93.1	86.2	82.8	89.7	96.6	89.7	82.8	69.0	82.8	96.6	89.7
Specificity (%)	88.9	100	100	88.9	61.1	94.4	94.4	77.8	94.4	72.2	77.8
AUC	0.946	0.960	0.960	0.908	0.839	0.954	0.941	0.807	0.921	0.906	0.900

*DAT* dopamine transporter, *dNDD* dopaminergic neurodegenerative disease, *ROC* receiver-operating characteristic, *DVR* distribution volume ratio, *BR* binding ratio, *COR* caudate-to-occipital lobe ratio, *POR* putamen-to-occipital lobe ratio, *SOR* striatum-to-occipital lobe ratio, *PCR* putamen-to-caudate ratio, *SBR* specific binding ratio, *SUV* standardized uptake value, *SUVmax* maximum SUV, *SUVmean* mean SUV, *AUC* area under the ROC curve

**Table 5:** Diagnostic accuracy of asymmetry of DAT quantitative values for dNDD according to ROC analysis

	Asymmetry on DVR			Asymmetry on BR			Asymmetry on SBR	Asymmetry on SUVmax	Asymmetry on SUVmean
	Caudate	Putamen	Striatum	Caudate	Putamen	Striatum			
Cutoff	5.31	4.85	5.22	41.1	15.7	4.31	12.0	4.44	5.64
Sensitivity (%)	65.5	69.0	62.1	24.1	51.7	75.9	75.9	72.4	58.6
Specificity (%)	83.3	61.1	83.3	94.4	83.3	72.2	83.3	55.6	77.8
AUC	0.772	0.647	0.728	0.387	0.678	0.734	0.757	0.680	0.649

*DAT* dopamine transporter, *dNDD* dopaminergic neurodegenerative disease, *ROC* receiver-operating characteristic, *DVR* distribution volume ratio, *BR* binding ratio, *SBR* specific binding ratio, *SUV* standardized uptake value, *SUVmax* maximum SUV, *SUVmean* mean SUV, *AUC* area under the ROC curve

The cutoffs of COR, POR, and SOR on BR were lower than those on DVR (1.34, 2.05, and 1.42 vs. 3.70, 2.62, and 2.88, respectively). Furthermore, COR on DVR and BR had superior sensitivity, while POR and SOR had superior specificity (sensitivities of 93.1% for COR on DVR and 96.6% for COR on BR; specificities of 100% for both POR and SOR on DVR and 94.4% for both POR and SOR on BR). Likewise, SBR, SUVmax, and SUVmean showed very high diagnostic accuracy for dNDD but were rather inferior to COR, POR, and SOR on DVR and POR and SOR on BR (AUCs: SBR, 0.921; SUVmax, 0.906; and SUVmean, 0.900). SBR had excellent specificity, whereas SUVmax and SUVmean had excellent sensitivity (specificity of 94.4% for SBR and sensitivities of 96.6% for SUVmax and 89.7% for SUVmean).

Striatal asymmetry on DVR and BR had moderate diagnostic accuracy, whereas putamen asymmetry was slightly inferior (AUCs of 0.728 for the striatum on DVR and 0.734 for the striatum on BR vs. 0.647 for the putamen on DVR and 0.678 for the putamen on BR). While caudate asymmetry on DVR had moderate diagnostic accuracy, that on BR was markedly worse, resulting in an inconsistency with many dNDDs below the cutoff (AUCs of 0.772 for the caudate on DVR vs. 0.387 for the caudate on BR). The diagnostic accuracy of asymmetry on SBR was moderate, while the accuracies of asymmetry on SUVmax and SUVmean were somewhat lower (AUCs of 0.757 for SBR vs. 0.680 for SUVmax and 0.649 for SUVmean).

### Correlations in DAT quantitative indices

**Fig. 3:**
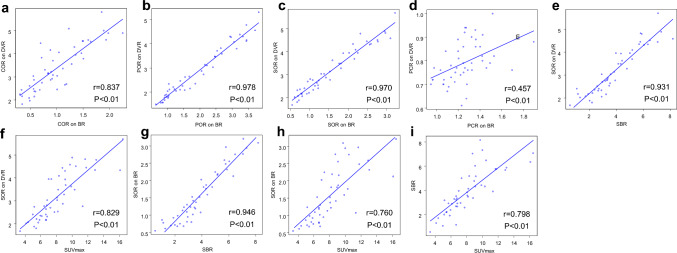
Correlations of DAT quantitative values in 47 patients. **a** COR on DVR and BR, **b** POR on DVR and BR, **c** SOR on DVR and BR, **d** PCR on DVR and BR, **e** SOR on DVR and SBR, **f** SOR on DVR and SUVmax, **g** SOR on BR and SBR, **h** SOR on BR and SUVmax, and **i** SBR and SUVmax

The correlations in DAT quantitative values are shown in Fig. 3. There were strong positive correlations between COR, POR, and SOR on DVR and those on BR (*r* = 0.837, *r* = 0.978, and *r* = 0.970, respectively; *P* < 0.01), whereas there was a weak correlation between PCR on DVR and that on BR (*r* = 0.457, *P* < 0.01). Strong positive correlations were found of SOR on DVR with SBR (*r* = 0.931) and SUVmax (*r* = 0.829) and of SOR on BR with SBR (*r* = 0.946) and SUVmax (*r* = 0.760) (*P* < 0.01). Similarly, a strong positive correlation was shown between SBR and SUVmax (*r* = 0.798, *P* < 0.01).

**Fig. 4:**
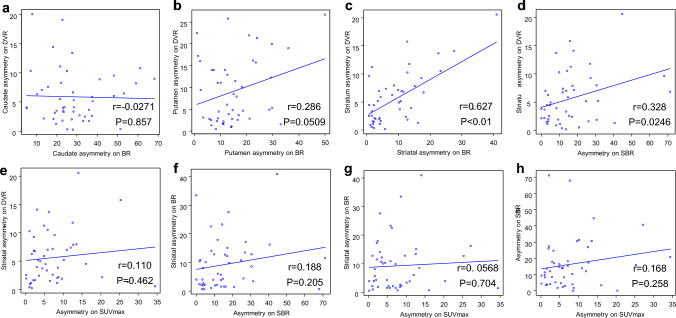
Correlations in the asymmetry of DAT quantitative values. **a** Caudate asymmetry on DVR and BR, **b** putamen asymmetry on DVR and BR, **c** striatal asymmetry on DVR and BR, **d** striatal asymmetry on DVR and SBR, **e** striatal asymmetry on DVR and SUVmax, **f** striatal asymmetry on BR and SBR, **g** striatal asymmetry on BR and SUVmax, and **h** SBR and SUVmax

The correlations in the asymmetries of the DAT quantitative values are shown in Fig. 4. There was a positive correlation between striatal asymmetry on DVR and that on BR (*r* = 0.627, *P* < 0.01). A weak but insignificant correlation was found between striatal asymmetry on DVR and asymmetry on SBR (*r* = 0.328, *P* = 0.0246). No correlation was seen in the asymmetries of the other DAT quantitative values.

## Discussion

We investigated the diagnostic ability of ^123^I-FP-CIT SPECT quantitative indices to differentiate dNDD from non-dNDD. Although it was not possible to include healthy controls in this retrospective study, we included non-dNDD patients, which are presumed to exhibit normal changes (i.e., not dopamine neurodegeneration). In clinical practice, it is necessary to differentiate non-dNDD from PD when determining treatment strategies. DVR and BR are quantitative indices of segmented striatal DAT uptake, but the clinical utility of BR was unclear. COR, POR, and SOR on DVR and POR and SOR on BR were significantly lower in dNDD than in non-dNDD, with AUCs of 0.941–0.960, indicating excellent utility for differentiating dNDD. COR on DVR and BR showed sensitivities of 93.1%–96.6%, while POR and SOR showed specificities of 94.4%–100%. Additionally, PCR on DVR exhibited an AUC of 0.908. In Oliveira et al., the average sensitivity and specificity of COR and POR in differentiating DLB from AD was 94% [13]. Cuberas-Borrós et al. reported that COR had a sensitivity of 77.5%, specificity of 100%, and AUC of 0.941, while POR had a sensitivity of 97.5%, specificity of 100%, and AUC of 0.999 in differentiating DIP or ET from PD, showing a similarly high diagnostic accuracy to our results [20]. Given the AUCs of 0.87 and 0.75 when using neuromelanin MRI in the substantia nigra and locus coeruleus for differentiating PD [29], as well as the sensitivity and specificity of 100% and 73.2%, respectively, when using brain perfusion SPECT in the occipital lobe for differentiating mild cognitive impairment (MCI) with Lewy bodies and MCI due to AD [30], DAT quantitative indices are reliable even when considered cross-sectionally.

We speculate that COR with high sensitivity would be useful for diagnosing probable dNDD and that the high specificity of POR and SOR would be useful for dNDD exclusion and ET or DIP diagnosis. Visual assessment is important because dNDDs sometime show quantitative values within the normal range despite reduced putamen accumulation. Because PCR has an AUC of 0.837 in differentiating parkinsonian syndrome (PS) including PD [18], PCR may be somewhat inferior to COR and POR, but the combination of SOR and PCR may be useful in the above case.

COR and PCR on BR showed AUCs of 0.839 and 0.807, respectively, indicating low diagnostic accuracy compared with the same indices on DVR and to POR and SOR on BR. Automated analysis of DVR has a success rate of 93.2% and agreement rate of 90.2% based on the imaging judgments of four physicians [31]. We identified no obvious misregistration of VOIs, regardless of DAT uptake. However, caudate uptake and VOI sometimes deviated in BR. Of the 29 dNDDs, 3 patients were slightly misregistered and 2 patients were markedly misregistered. Meanwhile, of the 18 non-dNDDs, only 1 patient was slightly misregistered. SPECT images are deformed with parameters that register simultaneously obtained CT image to MNI space to create a standard template for DVR analysis. If the original SPECT image is not correctly standardized to the template, registration can be based on the CT image. In contrast, in BR analysis, SPECT images are deformed with parameters that register the MRI image in different geometries from SPECT to MNI space. Therefore, preliminary co-registration of SPECT and MRI images of the same individual is needed. We speculate that these differences in technique may underlie the robustness of the VOI location in DVR, whereas, in BR, the diagnostic accuracy of COR and PCR decreases due to occasional misregistration of the caudate VOI. Anatomic standardization using CT-guided methods similar to DVR robustly differentiates PD [21], and the use of SPECT/CT images for template creation likely has advantages. Because the adaptive atlas used in BR enables accurate quantification of ^11^C-Pittsburgh compound B PET [27], it should also contribute to ^123^I-FP-CIT SPECT. However, with a large left/right difference in striatal uptake, one-sided striatal uptake may not conform to the mixed template with the adaptive atlas. The accuracy of the putamen VOI location was pretty high in BR, and POR on BR showed comparable diagnostic accuracy to that on DVR. The average variation of the values of the putamen is < 10% and the intra-class correlation coefficient is 98% in the various standard templates based on AAL atlas [19]. However, the values of the caudate and putamen are affected by spillover due to the partial volume effect [32]. Further research is required to determine the accuracy of DVR and BR analysis. On the other hand, COR was higher than POR on DVR, while POR was higher on BR, suggesting that the caudate VOI on DVR and putamen VOI on BR tend to contain more intermediate white matter with high DAT uptake. Moreover, COR, POR, and SOR were lower on BR than on DVR because DVR is the count ratio of the striatum to the occipital lobe, whereas BR is the count ratio of the striatum minus the occipital lobe to the occipital lobe.

SBR, SUVmax, and SUVmean were also significantly lower in dNDD, with AUCs of 0.921, 0.906, and 0.900, respectively. SBR showed an excellent specificity of 94.4%, and SUVmax and SUVmean showed sensitivities of 96.6% and 89.7%, respectively. SBR has reported specificities of 91.7%–97.0% and AUCs of 0.906–0.963 for differentiating dNDD [14, 18, 22–24], similar to our results. Furthermore, the SUVmax and SUVmean have reported sensitivities of 84.1%–87.5% and AUCs of 0.838–0.875 [22–24]. Compared with previous work, our values were somewhat higher, probably due to the use of the high-contrast OSCGM algorithm [33] and optimization of reconstruction parameters. SBR based on Bolt’s method has a large VOI surrounding the striatum, which reduces the partial volume effect and enables highly accurate quantification [14, 16]. However, because SBR assumes a constant theoretical striatal volume, it is significantly affected by sex and age-related atrophy [34]. SUV is unaffected by patient-specific factors, such as the administered radioactivity and body weight [22–24]. Meanwhile, SUV is affected by the degree of uptake in this method, where the VOI is determined by the maximum voxel value, and accurate normalization by body weight may be difficult for brain quantification. Verification of normalization in SUV by brain volume is desired. SBR, SUVmax, and SUVmean likely showed very high diagnostic accuracy for dNDD due to the above advantages but were slightly inferior to COR, POR, and SOR on DVR and POR and SOR on BR due to the above limitations.

Caudate and striatal asymmetries on DVR and striatal asymmetry on BR were significantly higher in dNDD than in non-dNDD, with AUCs of 0.728–0.772. In contrast, putamen asymmetries on DVR and BR were slightly lower, with AUCs of 0.647 and 0.678, respectively. Although striatal asymmetry demonstrated an AUC of 0.802 in differentiating PD, PS, and non-PS [18], our result is valid considering the different target patients. Moreover, DAT uptake decreases earliest in the posterior putamen, showing a distinct asymmetry, followed by the anterior putamen and finally progressing to the caudate in PD and PS [17, 35]. However, it should be noted that due to limitations of the DVR and BR software algorithm, it is not possible to analyze the putamen by dividing it into anterior and posterior parts. Caudate asymmetry on DVR exhibited higher diagnostic accuracy, presumably due to the aforementioned tendency of the caudate VOI to contain considerable intermediate white matter, which reduces noise and stabilizes values. On the other hand, the caudate asymmetry on BR was higher in non-dNDD, with an AUC of just 0.387, possibly due to caudate VOI misregistration. Because the striatum VOI includes the putamen, we assume that the influence was reduced, resulting in relatively high diagnostic accuracy. Asymmetry on SBR (AUC, 0.757), caudate and striatal asymmetry on DVR, and striatal asymmetry on BR were superior to the other parameters but had lower diagnostic accuracy than the quantification of DAT uptake itself.

Strong positive correlations were shown between COR, POR, and SOR on DVR and those on BR (correlation coefficients, 0.837–0.978). While DVR and BR differed in value and diagnostic accuracy for COR, their relative discrepancies were small, suggesting that these quantitative indices are subject-independent and comparable. Additionally, strong positive correlations were found of SOR on DVR with SBR or SUVmax, of SOR on BR with SBR or SUVmax (correlation coefficients, 0.760–0.946), and of SBR with SUVmax (correlation coefficient, 0.798). A significant strong positive correlation has been reported between SBR and SUVmax, with correlation coefficients of 0.752–0.795 [23, 24], similar to our results. Accordingly, DVR and BR can probably be compared with SBR and SUVmax, and the combination of DVR and BR with SUV and SBR, which is widely used in Japanese practice, should improve the diagnostic accuracy of dNDD.

Given the importance of severity assessment in PD treatment and care strategies, utilizing DAT quantitative indices for such assessments would be of great benefit. However, the indices on DVR and BR were comparable or higher as the HY stage progressed, contrary to our expectations. SBR, SUVmax, and SUVmean were lower with progression of HY stage, but none of the quantitative indices or their asymmetries between HY stages was significantly different. According to Eshuis SA, DAT uptake in the striatum had a moderate negative correlation with HY stage and with disease duration [36]. DAT uptake may be confounded not only by the severity of dNDD but also by the disease duration. Nevertheless, the disease duration was shorter for HY stage 3 than for HY stage 2, suggesting that the two factors had opposing effects on DAT quantitative values. In addition, because ^123^I-FP-CIT is used to diagnose dNDD in clinical practice, it is presumed that our patients were essentially in the early stage. Furthermore, although imaging changes were observed, symptoms had not yet appeared.

## Conclusion

COR, POR, and SOR on DVR and POR and SOR on BR are the most useful parameters for differentiating dNDD. These DAT quantitative indices can be compared with SBR and striatal SUV. Their comprehensive evaluation improves diagnostic accuracy.

## Appendix 1

See Table 1

**Table 1:** Definition of DVR, BR and SBR

Term	Common definition
DVR	Vt/Vr
BR SBR	Vt/Vr–1 = DVR–1

Ct and Cp generally represent tissue concentration and plasma concentration, respectively. Here, in DVR and BR, mean γ-ray count in the target tissue is applied to Vt and the one in the reference tissues to Vr. In SBR, Vt and Vr are assumed based on Bolt’s formula

*DVR* distribution volume ratio, *BR* binding ratio, *SBR* specific binding ratio, *Vt* volume of distribution, *Vr* reference volume of distribution

Vt = Ct/Cp
